# Behandlungserwartungen bei postoperativen Schmerzen

**DOI:** 10.1007/s00482-021-00575-0

**Published:** 2021-08-30

**Authors:** Julia Stuhlreyer, Regine Klinger

**Affiliations:** Universitätsklinikum Hamburg-Eppendorf, Martinistraße 52, 20246 Hamburg, Deutschland

**Keywords:** Messung der Behandlungserwartungen, Akutschmerz, Perioperative Situation, Placeboeffekt, Noceboeffekt, Measurement of treatment expectations, Acute pain, Perioperative setting, Placebo effect, Nocebo effect

## Abstract

**Hintergrund:**

Präoperative Behandlungserwartungen haben einen deutlichen Einfluss auf die postoperativen Schmerzen und Behandlungsergebnisse. Positive Erwartungen sind ein wichtiger Mechanismus von Placeboeffekten und negative Erwartungen ein wichtiger Mechanismus von Noceboeffekten.

**Fragestellung:**

Welchen Einfluss haben Behandlungserwartungen, wie werden diese im klinischen Setting erhoben und wie können diese Erkenntnisse in der klinischen Praxis umgesetzt werden?

**Material und Methoden:**

Es wurde eine Literatursuche für klinische Studien mit den Schlagwörtern „expectation“ AND („postoperative“ OR „surgery“) durchgeführt. Ausgewählt wurden alle aktuellen englischen und deutschen Artikel. Zusätzlich wurden die Literaturverzeichnisse der gefundenen Artikel untersucht und mit aufgenommen.

**Ergebnisse:**

Insgesamt 158 Artikel wurden gefunden, von denen 49 Artikel Erwartungen erheben und ein postoperatives Behandlungsergebnis einbeziehen. Die meisten Artikel untersuchen Erwartungen in der Baseline-Erhebung, um nachzuweisen, dass sich Gruppen in Gruppenvergleichen präoperativ nicht voneinander unterscheiden. Die Studien, die den Einfluss von Erwartungen prospektiv untersuchen, verwenden sehr unterschiedliche Messverfahren, um das Konstrukt „Erwartung“ zu erheben. Somit ist ein Vergleich zwischen den Studien schwer möglich. Es gibt wenige Studien, die untersuchen, ob und wie die Erwartungen perioperativ beeinflusst werden können, und die praxisrelevante Interventionen zu deren Veränderung entwickelt haben.

**Schlussfolgerung:**

Für eine fundierte Untersuchung der Behandlungserwartung sollten in klinischen Studien valide und reliable Messverfahren verwendet werden. Weitere Studien sollten sich mit Interventionsmöglichkeiten auseinandersetzen, damit Behandlungserwartungen auch in die klinische Standardbehandlung einbezogen werden können.

## Abstract (en)

**Background:**

Preoperative treatment expectations have a significant influence on postoperative pain and treatment outcomes. Positive expectations are an important mechanism of the placebo effect and negative expectations are an important mechanism of the nocebo effect.

**Objectives:**

What is the influence of treatment expectations, how are they assessed in the clinical setting, and how can the findings be implemented in clinical practice?

**Methods:**

A literature search was performed using the keywords “expectation” AND (“postoperative” OR “surgery”). All English and German articles were selected. In addition, the bibliographies of the articles found were examined and incorporated.

**Results:**

A total of 158 articles were found, 49 of which investigate expectations and include postoperative treatment outcomes. Most articles investigate expectations only at baseline to ensure that groups do not differ preoperatively. The studies that prospectively examine the influence of expectations apply very different measurement methods to investigate expectancy constructs. Thus, comparison across studies is difficult. There are few studies examining whether and how expectations can be influenced perioperatively, and who developed practice-relevant interventions to change them.

**Conclusion:**

Valid and reliable measurement tools should be applied in clinical trials for a more robust investigation of treatment expectations. Further studies should address possible intervention options so that treatment expectations can also be incorporated into standard clinical care.

Positive Behandlungserwartungen der Patienten führen zu geringeren postoperativen Schmerzen und beeinflussen das Behandlungsergebnis positiv [1]. Negative Erwartungen können dahingegeben das Behandlungsergebnis mindern oder sogar das Behandlungsziel verhindern [29]. Jedoch werden Erwartungen nicht einheitlich erhoben, sodass verschiedene Konstrukte zur Erwartung gemessen werden. Dennoch wäre es schon derzeit möglich, Studienerkenntnisse zur Optimierung der Behandlungsergebnisse in die Standardbehandlung einzubeziehen. Neuere Studien zeigen, dass Erwartungen präoperativ beeinflusst werden und so die Behandlungsergebnisse maximiert werden können [26].

Viele Patienten treffen ihre Entscheidung zu einer Operation aufgrund der Erwartung, dass sich ihr Gesundheitszustand dadurch verbessert. Patienten bewerten die zukünftigen Effekte der Operation und schätzen ein, ob und wie sie sich von der Operation erholen werden [29]. Das allein zeigt bereits den Einfluss der präoperativen Erwartungen von Patienten bei der Entscheidung für oder gegen eine Operation. Der Einfluss der Erwartungen bezüglich der Wirkung und der Effekte einer medizinischen Behandlung (sogenannte Behandlungserwartungen) endet nicht bei der Entscheidung, ob eine Operation überhaupt durchgeführt werden soll. Die Erwartung der Patienten beeinflusst über die Operation hinaus das Behandlungsergebnis maßgeblich. Positive Erwartungen können das Behandlungsergebnis positiv beeinflussen und sind ein wesentlicher Mechanismus des Placeboeffekts [20]. Negative Erwartungen der Patienten können den Behandlungserfolg dagegen verhindern oder unterdrücken und sind somit ein wesentlicher Mechanismus des Noceboeffekts. Die Einflüsse von Placebo- und Noceboeffekten sind sowohl in subjektiven (z. B. Schmerz) als auch in objektiven Parametern (wenige Studien; z. B. Blutmarker, Herzrate) nachweisbar [12, 17].

Der analgetische Effekt im postoperativen Schmerzmanagement kann durch Behandlungserwartungen beeinflusst werden

Der Einfluss von Kontextfaktoren konnte auch bei Operationen nachgewiesen werden [10]. Sihvonen et al. [25] haben herausgefunden, dass eine vorgetäuschte Kniearthroskopie („sham surgery“) bei Patienten mit Kniesymptomen, verursacht durch einen degenerativen medialen Meniskusriss und nicht durch Osteoarthritis, zu einem vergleichbar guten Behandlungsergebnis führte wie eine echte Kniearthroskopie. Generell erscheint es im Kontext von Operationen möglich, die Behandlungseffekte zu verstärken, indem die Erwartungen der Patienten mit einbezogen werden. So kann der analgetische Effekt im postoperativen Schmerzmanagement durch Behandlungserwartungen beeinflusst werden [20]. Dabei kann der therapeutische Nutzen von Analgetika durch positive Erwartungen verstärkt und durch negative Erwartungen gehemmt werden [6, 8].

## Entstehung von Erwartungen

**Figure 1:**
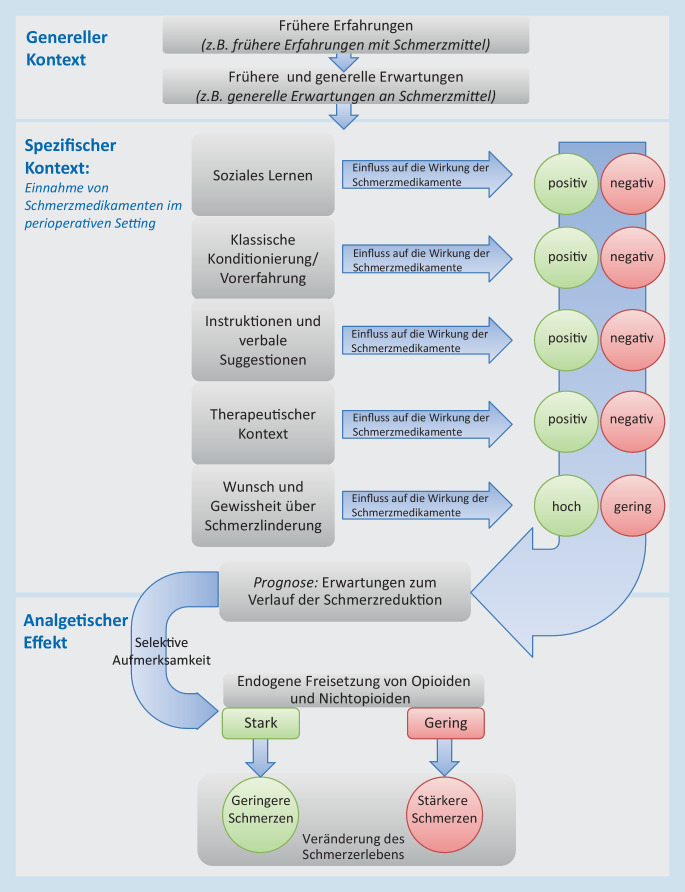

Obwohl Erwartungen der Patienten als Mechanismen in der psychologischen Forschung häufig untersucht werden, ist das dahinterstehende Konzept von Behandlungserwartungen noch nicht abschließend erforscht. Mehrere Kontextfaktoren sind bekannt, die Erwartungen maßgeblich beeinflussen und auf diesem Wege die endogene Schmerzhemmung anregen (Abb. 1). Erwartungen können entstehen und werden beeinflusst durch klassische Konditionierung, soziales Lernen, Instruktionen und den therapeutischen Kontext [14]. Diese Faktoren können jedoch oft nicht voneinander abgegrenzt werden. Daher ist auch möglich, dass in manchen Fällen die Erwartungen durch eine Kombination der Kontextfaktoren beeinflusst werden.

Die Mechanismen der Wirkung von Erwartungseffekten auf die Wirksamkeit von Schmerzmitteln sind in Abb. 1 dargestellt. Patienten haben generelle Einstellungen und Erwartungen basierend auf den erlebten Vorerfahrungen (z. B. „Ich nehme keine Schmerzmedikamente, weil sie mir langfristig schaden“). Diese Einstellungen können die Behandlungseffekte in einer spezifischen Behandlungssituation, etwa nach einer Operation, beeinflussen oder aber auch durch neue Erfahrungen revidiert werden. Die Einstellung zu Schmerzmedikamenten für diese spezifische Behandlungssituation kann sich ändern, wenn Patienten beispielsweise entsprechende Informationen erhalten („Postoperativ wirken Analgetika auch entzündungshemmend und das ist wichtig“). Die Einstellung (z. B. „Schmerzmedikamente werden mir helfen, weniger Schmerzen nach der Operation zu erleben“) löst positive Hoffnungen und Erwartungen an das postoperative Schmerzmanagement aus, die auch die Wahrnehmung von Schmerzmedikamenten selektiv beeinflussen. Im positiven Fall führt dies zu einer endogenen Freisetzung von Opioiden und Nichtopioiden, die den analgetischen Effekt verstärken und zu einer positiven Veränderung des Schmerzerlebens führen können. Demzufolge können Einflussfaktoren in der spezifischen Situation zu veränderten Erwartungen und Einstellungen führen, die Einfluss auf die postoperativ erlebten Schmerzen haben. Dieser Prozess kann postoperativ verstärkt werden, indem die Patienten auch im speziellen postoperativen Setting in ihren Erwartungen und ihrem Sicherheitsbedürfnis bestärkt werden.

### Klassische Konditionierung

Im Sinne der klassischen Konditionierung können Vorerfahrungen zu bewussten und unbewussten Behandlungserwartungen führen, die dann wiederum konditionierte Reaktionen auslösen [9]. So kann beispielsweise in einer medikamentösen Schmerzbehandlung die Einnahme eines Schmerzmedikaments (unkonditionierter Stimulus) zur Schmerzlinderung als unkonditionierte Reaktion (pharmakologische Wirkung) führen [7]. Hierbei kann ein eigentlich neutraler Stimulus (z. B. Aussehen des Schmerzmedikaments, Geruch von Schmerztropfen) durch assoziatives Lernen mit der Schmerzlinderung verknüpft werden und zum konditionierten Stimulus werden. Wenn die Patienten zukünftig das Medikament oder ein ähnlich aussehendes Medikament sehen, kann dies zu einer konditionierten Reaktion führen, die die tatsächliche Schmerzreduktion verstärkt. Der Effekt der klassischen Konditionierung kann mit mittleren bis großen Effekten bei akuten Schmerzen eingeschätzt werden [19].

### Soziales Lernen

Eine weitere Möglichkeit, Behandlungserwartungen zu beeinflussen, bietet das soziale Lernen. Durch Interaktionen und das Beobachten sozialer Situationen können Behandlungserwartungen der Patienten „übertragen“ und verstärkt werden [5, 15, 31]. Dies geschieht beispielsweise, wenn Patienten andere Patienten beobachten, die von einer spezifischen Behandlung profitieren. In dem Fall ist es wahrscheinlicher, dass auch der beobachtende Patient von der Behandlung profitiert. Soziales Lernen findet häufig im therapeutischen Kontext statt, da Patienten sich regelmäßig im sozialen Austausch mit Behandlern, Mitpatienten und Bekannten befinden. Experimentelle Studien zeigen, dass sowohl positive als auch negative Erwartungen der Patienten durch soziales Lernen entstehen können [2, 5].

### Instruktionen und verbale Suggestionen

Zusätzlich werden Behandlungserwartungen stark durch Instruktionen und verbale Suggestionen beeinflusst [11, 24]. Instruktionen unterstützen den Patienten dabei, die Behandlung und die darauffolgenden Konsequenzen zu verstehen. Instruktionen können genutzt werden, um realistische Erwartungen aufzubauen. Wenn unrealistische Erwartungen provoziert werden, können die Patienten enttäuscht sein, wenn diese nicht in Erfüllung gehen [13]. Die Effekte der verbalen Suggestionen wurden häufig untersucht und können als mittel bis groß eingeschätzt werden [19].

### Therapeutischer Kontext

Des Weiteren sind die Art der Intervention und der therapeutische Kontext relevant für die Entstehung von Behandlungserwartungen. Zum einen beeinflussen das Auftreten und äußere Erscheinungsbild des Behandlers die Erwartungen der Patienten, zum anderen werden größere Erwartungen in invasivere Verfahren gesetzt.

## Einfluss von Erwartungen auf postoperative Schmerzen und Lebensqualität

Um zu untersuchen, welche Auswirkungen die präoperativen Behandlungserwartungen der Patienten auf die postoperativen Schmerzen haben, sind klinische Studien unumgänglich.

Die Durchführung klinischer Studien ist unbedingt erforderlich

Der Einfluss von Erwartungen auf akuten Schmerz wird meist an gesunden Personen in einer kontrollierten Umgebung durchgeführt. Die Ergebnisse aus Laborstudien sind nicht ohne Weiteres auf Patienten und den klinischen Kontext zu übertragen. Deswegen ist es außerordentlich wichtig, dass klinische Studien durchgeführt werden, in denen Behandlungserwartungen im Zusammenhang mit akuten postoperativen Schmerzen und den Behandlungsergebnissen untersucht werden.

### Zusammenhang von Erwartungen mit postoperativen Schmerzen

Die Untersuchung und Einbeziehung der Erwartungen ist bei manchen Operationen weit verbreitet. In Bezug auf Knie- und Hüftendoprothetik gehören Erwartungen zu den am meisten untersuchten prognostischen Faktoren [16]. Eine Metaanalyse zeigt, dass der Zusammenhang zwischen der positiven präoperativen Erwartung und der postoperativen patientenorientierten Zielvariablen (z. B. Schmerz) bei Knie- und Hüftendoprothetik zwar klein, aber robust ist [16].

Sobol-Kwapinska et al. [26] haben in einer Metaanalyse den Zusammenhang von psychologischen Korrelaten und postoperativen Schmerzen untersucht. Von den untersuchten präoperativen psychologischen Variablen zeigte Schmerzkatastrophisierung den stärksten Zusammenhang mit postoperativen Schmerzen, gefolgt von Optimismus, Schmerzerwartungen, Neurotizismus, Angst als Charaktereigenschaft („trait“) und als Zustand („state“), negativen Auswirkungen und Depressionen. Also verstärken vor allem präoperativ katastrophisierende Gedanken bezüglich der postoperativen Schmerzen die erlebten postoperativen Schmerzen. Andererseits sind postoperative Schmerzen geringer, wenn die Patienten präoperativ optimistisch eingestellt sind. Deswegen ist für eine bessere postoperative Schmerzverarbeitung wichtig, dass negative Aspekte der Situation nicht übertrieben werden und positive Aspekte der Zukunft hervorgehoben werden [26].

### Einfluss präoperativer Erwartungen auf die Lebensqualität

Auch andere Behandlungsergebnisse wie die Lebensqualität können durch präoperative Erwartungen beeinflusst werden. In einer Metaanalyse von Auer et al. [1] wurde der Einfluss präoperativer Erwartungseinstellungen auf die postoperative Lebensqualität untersucht. Eine Subgruppenanalyse zeigt, dass die präoperativen Erwartungen und die generelle Lebensqualität mittelmäßig, Erwartungen und physische Lebensqualität gering und Erwartungen und mentale Lebensqualität mittelmäßig miteinander korrelieren. Der Zusammenhang zwischen Erwartungen und genereller Lebensqualität ist nicht von der Art der Operation, dem Erwartungskonstrukt oder dem verwendeten Messinstrument, um Erwartungen zu erheben, abhängig. In Bezug auf die mentale Lebensqualität ergab die Subgruppenanalyse, dass das zugrunde liegende Erwartungskonzept einen signifikanten Einfluss hat. Der Zusammenhang war am stärksten, wenn Optimismus als zugrunde liegendes Konstrukt verwendet wurde, und am kleinsten, wenn Pessimismus oder Erholungserwartungen untersucht wurden [1].

## Messinstrumente für präoperative Erwartungen

Wenn Behandlungserwartungen in verschiedenen klinischen Studien erhoben werden, ist nicht garantiert, dass jeweils dieselben Erwartungen erhoben werden. Um Studien sinnvoll miteinander vergleichen zu können und sicherzustellen, dass auch tatsächlich vergleichbare Erwartungen erhoben werden, ist relevant, dass validierte und reliable Messinstrumente eingesetzt werden. Der Einsatz qualitativ höherwertiger Fragebögen zeigt, dass der Zusammenhang zwischen Erwartungen und patientenorientierten Endpunkten stärker ist [16]. Bisher werden jedoch häufig keine validierten Fragebögen oder validierten Einzelskalen zur Erhebung von Erwartungen eingesetzt. Die verwendeten nicht validierten Fragebögen oder Einzelfragen unterscheiden sich nicht nur inhaltlich, sondern auch in ihren Antwortmöglichkeiten.

Bei der Erhebung von Behandlungserwartungen im Kontext von Operationen ist es relevant zu entscheiden, worauf sich die Erwartung genau beziehen soll. Hier lassen sich unter anderem unterscheiden:

- Allgemeine Behandlungserwartungen (z. B. „Ich erwarte nur Gutes von medizinischen Behandlungen“)
- Allgemeine operationsspezifische Erwartungen (z. B. „Ich erwarte nur Gutes von meiner TEP-Operation“)
- Operationstechnikspezifische Erwartungen (z. B. „Ich erwarte, dass die Operateure den Eingriff an meinem Knie qualitativ hochwertig durchführen werden“)
- Erwartungen bezogen auf die Symptomatik, die zur Operation führte (z. B. „Ich erwarte, dass ich mein Knie nach der Operation wieder schmerzfrei bewegen kann“)
- Erwartungen bezogen auf den postoperativen Wundschmerz (z. B. „Ich erwarte, dass die postoperativen Schmerzen, die durch den operativen Eingriff an sich verursacht werden, erträglich sein werden“)
- Erwartungen bezogen auf die symptomspezifische kurz- und langfristige postoperative Situation (z. B. „Ich erwarte, dass meine Knieschmerzen nach der Operation im Krankenhaus erträglich sind und auch danach zu Hause gar nicht mehr auftreten werden“)

Die erfragten Erwartungen bezogen auf die symptomspezifischen kurz- und langfristigen postoperativen Situationen sollten auf den erwarteten postoperativen Schmerz, die körperliche Funktionskapazität und den Schmerzmittelverbrauch bezogen sein. Die Wahl der verwendeten Fragebögen hängt primär von der zu untersuchenden Fragestellung ab. In Tab. 1 findet sich eine Übersicht gängiger Skalen und Instrumente, die verwendet werden, um Erwartungen im perioperativen Kontext zu erheben.

**Table 1:** 

Instrument oder Skala	Inhalt	Vorteil	Nachteil
**Allgemeine Behandlungserwartungen**			
*Validierte Fragebögen*			
Stanford Expectations of Treatment Scale (SETS; [30])	Erhebt positive und negative Behandlungserwartungen	Validierte Skala	Behandlung ist an sich nicht näher definiert, sodass nicht zwischen den verschiedenen Behandlungen unterschieden wird (z. B. Operation, Medikamente)
	Beispielitem: „Diese Behandlung wird komplett effektiv sein.“	Für alle Operationen (universell) einsetzbar	
		Schnelle Durchführung	
Life Orientation Test (LOT[-R])	Erhebt Optimismus vs. Pessimismus	Validierte Skala	Erhebt generelle Erwartungen und keine Behandlungserwartungen
	Beispielitem: „Auch in ungewissen Zeiten erwarte ich normalerweise das Beste.“	Sehr häufig eingesetzt bei der Erfassung von dispositionellem Optimismus	
Revised Illness Perception Questionnaire (IPQ-R)	Erhebt kognitive Krankheitspräsentationen, Ängstlichkeit, Depressivität, allgemeine Selbstwirksamkeitserwartung und Optimismus	Validierte Skala	Erhebt keine allgemeinen oder spezifischen Behandlungserwartungen
	Beispielitem: „Meine Krankheit wird lange Zeit andauern.“	Weit verbreitete Anwendung	
BREAST‑Q	Patienteneinschätzungen, um Ergebnisse einer Brustoperation zu erheben	Validierte Skala	Ausschließlich einsetzbar für Brustoperationen
		Erfasst Erwartungen in verschiedenen Bereichen (z. B. Unterstützung des medizinischen Personals, Schmerzen)	Erwartungen sind ein Modul (von 6)
		Perioperative Anwendung (Instrument wird prä- und postoperativ ausgefüllt)	
Knee Society Scoring System	Erhebt primär die Funktion der Knieprothese und die Funktionskapazitäten der Patienten nach einer Knieendoprothesenoperation	Validierte Skala	Ausschließlich einsetzbar für Knieendoprothesenoperationen
	Beispielitem: „Erwarten Sie von Ihrer Kniegelenkersatz-Operation eine Linderung Ihrer Knieschmerzen?“	Perioperativer Einsatz (Instrument wird prä- und postoperativ ausgefüllt)	Erwartungen sind ein Modul (von 5)
*Nichtvalidierte Fragebögen*			
„Generic rating scale for previous treatment experiences, treatment expectations, and treatment effects“ (GEEE; [22])	Screening-Tool für eine allgemeine Bewertung und Quantifizierung von Behandlungserwartungen und deren Auswirkung auf klinische Ergebnisse	Für viele Behandlungen (universell) einsetzbar	Noch nicht abschließend validiert
	Beispielitem: „Wie viel Verbesserung der (Primäres Outcome) erwarten Sie durch die Behandlung (Name der Behandlung)?“	Ermöglicht den Vergleich zwischen verschiedenen Behandlungen	Wurde nicht für perioperative Behandlungserwartungen entwickelt
Selbstentwickelte Fragebögen oder Einzelskalen	Inhalt unterscheidet sich je nach Forschungsfrage	Können flexibel an die Forschungsfrage angepasst werden	Sind nicht als valide oder reliabel nachgewiesen, nur in Teilen des Instruments
	Beispielitems: „Was erwarten Sie, wie stark werden Ihre Schmerzen nach der Operation sein?“		Ergebnisse aus verschiedenen Studien können nicht miteinander verglichen werden
**Symptomspezifischer Fragebogen**			
Noch nicht abschließend entwickelt	Sollte flexibel an verschiedene Operationen anpassbar sein		
	Sollte sich auf symptomspezifische Behandlungserwartungen beziehen (z. B. Schmerzen)		
	Sollte prä- und postoperativ angewendet werden		
	Könnte sich inhaltlich am Vorgang von Schmitz et al. [24] orientieren		
	Sollte änderungssensitiv sein		
	Könnte bei einheitlicher Systematik der Fragestruktur Erwartungen zu unterschiedlichen medizinischen Behandlungen vergleichen		

### Allgemeine Behandlungserwartungen.

Zur Erhebung genereller präoperativer Behandlungserwartungen eignet sich die validierte Stanford Expectations of Treatment Scale (SETS) von Younger et al. [30]. Die Autoren konnten mit dem Messinstrument ermitteln, dass Behandlungserwartungen bei Patienten, die operiert werden, 12–18 % der Ergebnisvarianz vorhersagen. Dieses Messinstrument ermöglicht einerseits, den Einfluss der Behandlungserwartungen in Studien statistisch abzugrenzen. Andererseits ist es mit der Skala möglich, im klinischen Alltag Patienten mit negativen Erwartungen zu identifizieren und entsprechend präoperativ zu unterstützen. Dieser Fragebogen wurde für alle Operationen entwickelt, sodass er nicht nur bei einer speziellen Behandlung eingesetzt werden kann. Er bezieht sich allerdings lediglich auf die positiven und negativen Erwartungen einer Behandlung, berücksichtigt aber keine spezifischen Symptome.

Ein neueres Instrument, das derzeit noch auf Validität und Reliabilität überprüft wird, ist die „Generic rating scale for previous treatment experiences, treatment expectations, and treatment effects“ (GEEE; [22]). Die GEEE lässt sich der Behandlung entsprechend anpassen, sodass die Skala sowohl im klinischen als auch im experimentellen Setting eingesetzt werden kann. Sie bezieht inhaltlich generelle Behandlungserwartungen und Vorerfahrung zur Behandlung ein und bietet die Möglichkeit, die Effekte regelmäßig zu überprüfen.

Auer et al. [1] empfehlen basierend auf ihrer Metaanalyse die Verwendung des Life Orientation Test (LOT[-R]) und Revised Illness Perception Questionnaire (IPQ-R) als Erwartungsinstrument, da diese weitverbreitete und validierte Erhebungsinstrumente sind. Jedoch bezieht sich der LOT(-R) nicht auf spezifische Behandlungserwartungen, sondern erhebt Optimismus vs. Pessimismus als Persönlichkeitsvariablen. Auch der IPQ‑R erhebt keine spezifischen Behandlungserwartungen, sondern erfasst kognitive Krankheitspräsentationen, Ängstlichkeit, Depressivität, allgemeine Selbstwirksamkeitserwartung und Optimismus. Diese Unterschiede in der Möglichkeit, Erwartungen zu erheben, können zu kontroversen Studienergebnissen führen.

Es gibt derzeit noch nicht viele valide und reliable Messinstrumente, die für spezifische Operationen Erwartungen einbeziehen. Für die Brustrekonstruktion nach Brustkrebs gibt es den Fragebogen BREAST‑Q, der ein Modul für die Erfassung der Erwartungen beinhaltet. Die Erwartungen beziehen sich beispielsweise auf den Schmerz oder die Verarbeitung der Operation [21]. Für Knieoperationen gibt es das Knee Society Scoring System [18], das die Erwartungen der Behandlung spezifisch für Schmerzen, täglichen Aktivitäten, prä- und postoperative Aktivitäten und die Erfüllung der Operationserwartungen erfragt. Diese seltene Einbindung von Erwartungen in operationsspezifische Fragebögen zeigt, dass Erwartungen noch nicht als fester Bestandteil in der Fragebogenkonstruktion und Behandlung etabliert sind.

### Symptomspezifische Behandlungserwartungen.

Um symptomspezifische Behandlungserwartungen (etwa in Bezug auf postoperative Schmerzen) zu erheben, gibt es noch kein abschließend validiertes Messinstrument. Daher werden derzeit verschiedene Einzelfragen verwendet, um postoperative Schmerzerwartungen zu erheben. Sinnvoll für den Bereich „Schmerz“ wäre eine Erhebung durch ein Messinstrument, das universell eingesetzt und somit für verschiedene Operationen flexibel angepasst werden kann. Wichtig ist, dass die Erwartungen präoperativ erhoben werden und sich auf die relevanten postoperativen Symptome beziehen, beispielsweise auf postoperative Schmerzen in Ruhe, postoperative Schmerzen in Aktivität und Wundschmerzen. Um eine genauere Auswertung zu ermöglichen, sollte eine numerische Rating-Skala von 0 bis 10 als Messskala verwendet werden. Ausgehend vom aktuellen Schmerz sollte der erwartete, das heißt prognostizierte postoperative Schmerz und somit auch die erwartete Schmerzdifferenz erhoben werden (vgl. [24]). Die Erwartungen sollten nicht nur präoperativ erfasst, sondern auch postoperativ überprüft werden. Mit diesem Vorgehen lassen sich auch andere symptomspezifische Erwartungen erheben, beispielsweise physische Funktionskapazität oder Medikamentengebrauch. Dieses Vorgehen zeigte sich auch in Studien zum analgetischen Placeboeffekt nach Scheinopioidgabe als änderungssensitiv [24]. Deswegen wird dieses Messverfahren derzeit weiterentwickelt, an das postoperative Setting angepasst und validiert.

## Interventionen zur Beeinflussung von Behandlungserwartungen im klinischen Kontext

Der Zusammenhang zwischen präoperativen Erwartungen und postoperativen Schmerzen wird in den meisten Fällen prospektiv untersucht und die Behandlungserwartungen werden nicht bewusst und gezielt beeinflusst. Dass präoperative Erwartungen die postoperativen Schmerzen und das Behandlungsergebnis beeinflussen, ist mittlerweile gut belegt. Für die zukünftige Forschung ist es notwendig und wichtig, Möglichkeiten zu ermitteln, wie die Behandlungserwartungen beeinflusst werden können. Es gibt jedoch derzeit nur sehr wenige Studien, die mögliche perioperative Interventionen entwickeln und wissenschaftlich testen. Benson et al. [3] konnten bei Patientinnen, die einer brusterhaltenden Operation unterzogen wurden, ergänzend zur Opioidanalgesie mithilfe einer kurzen psychologischen Erwartungsintervention postoperative Schmerzen reduzieren. Sie verglichen den Einfluss positiver Suggestionen mit neutralen Suggestionen und untersuchten zusätzlich den Einfluss von Sham-Akupunktur. Positive Suggestionen reduzierten die postoperativen Schmerzen deutlich, unabhängig davon, ob Sham-Akupunktur durchgeführt wurde oder nicht. In einer anderen Studie unterstützten Rief et al. [23] Patienten, die einen Koronararterienbypass erhielten, durch ein spezielles perioperatives Erwartungsmanagement und verglichen sie sowohl mit einer Patientengruppe, die zwar therapeutisch unterstützt, aber nicht speziell bezüglich der Erwartungen behandelt wurde, als auch mit einer Standardtherapiegruppe. Subjektive Parameter, wie Beeinträchtigung und Lebensqualität 6 Monate nach der Operation, aber auch objektive Immunparameter konnten durch die spezielle psychologische Intervention verbessert werden [23]. Die Ergebnisse zeigen die Relevanz der Einbeziehung der Erwartung in die Behandlung.

Es fehlen innovative Interventionen, die Erwartungen einbeziehen

Ein weiteres Hilfsmittel, um Behandlungserwartungen zu beeinflussen, sind digitale Anwendungen. Personalisierte E‑Health-Methoden konnten bei Bauchoperationen die Rückkehr zu normalen Aktivitäten beschleunigen [28]. In einer eigenen Studie an 120 Patienten, die eine Knietotalendoprothese erhalten haben, wurde bereits gezeigt, dass mithilfe einer speziellen Tablet-basierten Applikation im Zusammenhang mit patientenorientierten Schmerzvisiten die postoperativen Schmerzen und der postoperative Opioidkonsum reduziert werden konnten [27]. In dieser Studie konnten zusätzlich auch die Erfüllung der Erwartungen und die Zufriedenheit mit der Operation gesteigert werden. Durch den individualisierten Einsatz technischer Tools kombiniert mit einer positiven Arzt-Patienten Interaktion wurde hier das postoperative Schmerzmanagement optimiert [27].

## Zukünftige Forschung

Klinische Studien im perioperativen Setting verfolgen zumeist das Ziel, das Operationsergebnis zu verbessern. Wird das Konstrukt „Erwartung“ einbezogen, zeigt sich, dass die Operationsergebnisse nicht nur von der Operationstechnik oder einer veränderten Medikation abhängig sind, sondern auch von der Veränderung der Kontextfaktoren. Dazu gehört, dass realistische und konkrete Erwartungen der Patienten in die Behandlung einbezogen werden. Aus diesem Grund ist es wichtig, weitere hilfreiche Interventionen zu entwickeln und wissenschaftlich zu untersuchen, um die Behandlungserwartungen zu beeinflussen und dadurch postoperative Schmerzen und andere relevante Behandlungsergebnisse positiv zu beeinflussen.

In zukünftigen Studien sollte untersucht werden, mit welchen Interventionen die Behandlungserwartungen beeinflusst werden können. Darüber hinaus ist es wichtig, Personengruppen zu identifizieren, die von der Beeinflussung der Behandlungserwartung profitieren, um damit das postoperative Behandlungsergebnis positiv beeinflussen zu können [4].

## Fazit für die Praxis

- Sowohl positive als auch negative Erwartungen sollten vor der Operation erfragt werden, am besten mit einem validierten Fragebogen und in einem persönlichen Gespräch.
- Das persönliche Gespräch sollte kompetent und empathisch durchgeführt werden, am besten durch speziell geschultes Personal.
- Lassen Sie den Patienten Erwartungen so konkret wie möglich ausformulieren.
- Es sollte eingeschätzt werden, wie realistisch die Erwartungen sind. Realistische Erwartungen werden verstärkt, um den Placeboeffekt zu maximieren.
- Bei unrealistischen Erwartungen sollte ein aufklärendes Gespräch geführt werden, um Erwartungen zu relativieren.
- Auf die Formulierung möglicher negativer Informationen (beispielsweise in Bezug auf Nebenwirkungen) sollte geachtet werden. Ein balancierter Vortrag ist wichtig. das heißt, die Anzahl negativer Informationen sollte mit der gleichen Anzahl positiver Informationen ausbalanciert werden, sodass Noceboeffekte möglichst vermieden werden können.
- Nach der Operation sollten die positiven Erwartungen für den weiteren Verlauf verstärkt werden.
