# Erratum zu: Behandlungserwartungen bei postoperativen Schmerzen

**DOI:** 10.1007/s00482-022-00625-1

**Published:** 2022-02-16

**Authors:** Julia Stuhlreyer, Regine Klinger

**Affiliations:** Universitätsklinikum Hamburg-Eppendorf, Martinistraße 52, 20246 Hamburg, Deutschland

**Erratum zu:**

**Schmerz**

10.1007/s00482-021-00575-0

Der Artikel *Behandlungserwartungen bei postoperativen Schmerzen* von Julia Stuhlreyer und Regine Klinger wurde ursprünglich am 30. August 2021 ohne „Open Access“ online auf der Internetplattform des Verlags publiziert. Die Autoren haben sich jedoch nachträglich für eine „Open Access“-Veröffentlichung entschieden. Das Urheberrecht des Artikels wurde deshalb am 17. Januar 2022 in © Der/die Autor(en) 2021 geändert. Der Artikel wird nun unter der Creative Commons Namensnennung 4.0 International-Lizenz veröffentlicht, welche die Nutzung, Vervielfältigung, Bearbeitung, Verbreitung und Wiedergabe in jeglichem Medium und Format erlaubt, sofern Sie den/die ursprünglichen Autor(en) und die Quelle ordnungsgemäß nennen, einen Link zur Creative-Commons-Lizenz beifügen und angeben, ob Änderungen vorgenommen wurden.

Die in diesem Artikel enthaltenen Bilder und sonstiges Drittmaterial unterliegen ebenfalls der genannten Creative-Commons-Lizenz, sofern sich aus der Abbildungslegende nichts anderes ergibt. Sofern das betreffende Material nicht unter der genannten Creative-Commons-Lizenz steht und die betreffende Handlung nicht nach gesetzlichen Vorschriften erlaubt ist, ist für die oben aufgeführten Weiterverwendungen des Materials die Einwilligung des jeweiligen Rechteinhabers einzuholen.

Weitere Details zur Lizenz entnehmen Sie bitte der Lizenzinformation auf http://creativecommons.org/licenses/by/4.0/deed.de.

